# Quando o Estado bate à porta e avisa: “agora é com você”

**DOI:** 10.1590/0102-311XPT186225

**Published:** 2026-03-09

**Authors:** Marina Belmiro Gomes de Souto, Joviana Quintes Avanci Pina, Fernanda Mendes Lages Ribeiro

**Affiliations:** 1 Instituto Nacional de Saúde da Mulher, da Criança e do Adolescente Fernandes Figueira, Fundação Oswaldo Cruz, Rio de Janeiro, Brasil.

Em *Saúde Mental e Relações Sociais - Desnorteamento, Aquilombação e Antimanicolonialidade*, Emiliano de Camargo David ^1^ nos convoca a abandonar redes institucionais de cuidado que, embora consolidadas, muitas vezes reproduzem exclusões. Seu livro não é apenas uma crítica às práticas em saúde mental; é um convite a se perder e reinventar modos de existir e cuidar. É leitura para quem aceita sair do conforto conceitual e ouvir vozes que ecoam das brechas do sistema, dos abrigos, dos terreiros e dos quintais que nunca couberam no manicômio.

A obra chega em um momento em que, apesar dos avanços da Reforma Psiquiátrica, persistem lacunas que comprometem seus princípios. Situações concretas, como o desligamento compulsório de jovens ao completarem 18 anos em instituições de acolhimento, a insuficiência de dispositivos comunitários e a permanência de práticas de segregação, revelam que a promessa de cidadania plena ainda não se concretizou. David aborda o abandono institucional, o racismo estrutural e a forma como a fragilidade das redes sociais expõe sujeitos a vulnerabilidades extremas quando dependem da tutela estatal.

O livro organiza-se em quatro capítulos. Os três primeiros desenvolvem os eixos conceituais centrais - desnorteamento, aquilombação e antimanicolonialidade. O capítulo sobre desnorteamento propõe uma ruptura conceitual, afirmando loucura e negritude como forças que desalojam fixações patológicas e raciais. O capítulo sobre aquilombação defende territórios insurgentes de cuidado, inspirados na tradição quilombola e sustentados em práticas comunitárias horizontais. Já o capítulo sobre antimanicolonialidade articula crítica racial, descolonização epistemológica e ruptura com o silenciamento de saberes não brancos. Por fim, o quarto capítulo apresenta experiências concretas que materializam tais conceitos no cotidiano da rede de atenção psicossocial, funcionando como elo entre a densidade teórica e sua aplicação.

Há potência no conceito de desnorteamento quando o aproximamos da condição de adolescentes em desligamento institucional, relação que David não formula diretamente. Aos 18 anos, o Estado brasileiro bate à porta e avisa: “Agora é com você”. No papel, a maioridade garante direitos civis; na vida, reinaugura-se o abandono. Como se autonomia fosse decreto, maturidade fosse questão de idade e 18 anos apagassem uma história inteira de ausências.

Castel ^2^ descreve sujeitos que não perdem vínculos porque nunca os tiveram: os desfilados, que caminham sem rede, sem mapa, sem bússola. David dá corpo a essa sensação sem romantizar ou individualizar o sofrimento: não se trata de falhas morais, mas de falências políticas. A autonomia jurídica, nesse contexto, é ficção cruel, como argumenta Sarmento ^3^, pois se sustenta na negação dos suportes sociais necessários à vida adulta.

Nesse percurso, é impossível não evocar vozes brasileiras que já denunciavam a colonialidade do poder e do saber. Lélia Gonzalez ^4^, ao articular racismo e sexismo no conceito de *amefricanidade*, lembra que cuidado e política só podem ser compreendidos a partir da experiência concreta dos corpos negros na diáspora. Sueli Carneiro ^5^, com o conceito de dispositivo de racialidade, mostra como o racismo estrutura não apenas relações interpessoais, mas também tecnologias institucionais de exclusão. O desligamento institucional, nesse sentido, é efeito de um dispositivo que racializa a exclusão e fabrica abandonos.

Trazer essas autoras não é apenas opção bibliográfica, mas gesto político de aquilombamento do pensamento. Se Fanon ^6^ e Mbembe ^7^ abrem caminhos no debate anticolonial, é nas formulações de intelectuais negras brasileiras que se encontra o chão para repensar políticas públicas no Brasil. Ao lado da aquilombação de David ^1^, a *amefricanidade* de Gonzalez ^4^ e o dispositivo de racialidade de Carneiro ^5^ oferecem ferramentas para desnudar como a legislação adultocêntrica e o Estado neoliberal operam sobre corpos jovens e negros. São contribuições que deslocam o olhar, tensionam narrativas universais e reafirmam a saúde mental como disputa política e histórica.

Se o Estado expulsa, o quilombo acolhe. O conceito de aquilombação estabelece uma ponte inovadora entre a tradição quilombola de resistência e as lutas contemporâneas por justiça em saúde mental. Aquilombar-se é reinventar espaços de cuidado fora da lógica manicomial, da tutela estatal e da normatividade branca. É forjar redes onde antes havia muros.

Inspirado em epistemologias negras e práticas de cuidado ancestral, o autor reposiciona o cuidado como gesto político e relacional. É possível aquilombar um Centro de Atenção Psicossocial (CAPS)? Um coletivo de jovens egressos? Um projeto social na periferia? Segundo David ^1^, sim, desde que se subverta o centro e se valorize o que cresce nas bordas. Essa perspectiva dialoga com Bourdieu ^8^ ao pensar o capital social não como moeda acumulada em redes hegemônicas, mas como potência organizadora da vida em comunidades que sobrevivem à margem. Ao aquilombar o cuidado, David ^1^ aponta que é no território simbólico, no afeto e na ancestralidade que se gesta a autonomia real, aquela que não se decreta, mas se cultiva.

No contexto do desligamento institucional, a aquilombação surge como ferramenta conceitual valiosa para pensar estratégias de autonomia que não reproduzam lógicas excludentes. O território aquilombado é espaço de produção de subjetividades alternativas, onde o cuidado em saúde mental pode ser repensado a partir de práticas comunitárias e horizontais.

Talvez a maior ousadia do livro seja afirmar que cuidar é, antes de tudo, desobedecer. Ao recusar saberes coloniais que patologizam corpos racializados e silenciam saberes não brancos, David ^1^ reivindica uma saúde mental antimanicomial e anticolonial, não por ideologia, mas por coerência histórica.

Em diálogo com Fanon ^6^ e Mbembe ^7^, a antimanicolonialidade não aparece como metáfora, mas como método, ética e epistemologia. Descolonizar o cuidado exige reencantar a escuta, desconstruir o sujeito universal da psicologia e reconectar sofrimento à história da escravidão, do racismo estrutural e das políticas de controle.

O estigma, conforme Goffman ^9^, é a marca que transforma pessoas em categorias. No acolhimento institucional, essa marca é visível: o “egresso” carrega o rótulo de “problema social”. O que David ^1^ mostra é que somente uma prática anticolonial pode desativar esse dispositivo.

Apesar da potência conceitual, a articulação entre desnorteamento, aquilombação e antimanicolonialidade poderia ter costura mais clara entre teoria e prática, sobretudo quanto à aplicação em políticas públicas. A proposta antimanicomial é urgente, mas ainda carece de caminhos concretos.

A obra, no entanto, acende perguntas essenciais. Seu desafio talvez seja transformar essas perguntas em estratégias possíveis sem perder a radicalidade que as originou. *Saúde Mental e Relações Sociais - Desnorteamento, Aquilombação e Antimanicolonialidade* ^1^ é livro que deveria circular nas rodas de CAPS, Conselhos Tutelares, formações profissionais, corredores ministeriais e bancas acadêmicas. Obriga-nos a olhar para aquilo que a política pública não quer ver: a existência concreta de sujeitos institucionalizados pela ausência, silenciados pelo racismo e descartados pela lógica adultocêntrica.

No campo da Saúde Coletiva, David ^1^ atualiza a radicalidade do cuidado como ato político. Para quem pesquisa o desligamento institucional, como esta autora, o livro é lente necessária para desnaturalizar o modo como o Estado transita da tutela ao abandono, chamando esse gesto de “emancipação”.

Tal como Deleuze & Guattari ^10^ falaram de linhas de fuga como potência criadora, Emiliano David ^1^ nos oferece três linhas desnorteamento, aquilombação e antimanicolonialidade, não como conceitos fechados, mas como vetores de reexistência. A saúde mental brasileira precisa de outras rotas. Este livro é guia, é bússola.
